# The Association of Left Atrial Appendage Morphology to Atrial Fibrillation Recurrence After Radiofrequency Ablation

**DOI:** 10.3389/fcvm.2021.677885

**Published:** 2021-08-12

**Authors:** Shiyu Gong, Jian Zhou, Bingyu Li, Sheng Kang, Xiaoye Ma, Ying Cai, Yang Guo, Rui Hu, Xumin Zhang

**Affiliations:** ^1^Department of Cardiovascular Medicine, Shanghai East Hospital, School of Medicine, Tongji University, Shanghai, China; ^2^Department of Cardiology, Shanghai Tenth People's Hospital, School of Medicine, Tongji University, Shanghai, China; ^3^Department of Laboratory, Taiyuan Hospital Health Center for Woman and Children, Taiyuan, China

**Keywords:** atrial fibrillation, recurrence, radiofrequency ablation, left atrial appendage morphology structure, recurrence risk

## Abstract

**Objective:** The probability of late recurrent atrial fibrillation (AF) after radiofrequency ablation (RFA) has not yet been fully clarified. This study aims to study the association of left atrial appendage (LAA) morphology with AF recurrence after RFA.

**Methods:** We retrospectively enrolled 84 patients (24 patients had persistent AF, 60 patients had paroxysmal AF) who underwent RFA in Shanghai East Hospital from June 2014 to May 2018. The mean follow-up of these patients was 618.6 days. According to preoperative transesophageal echocardiography (TEE), the morphology feature of LAA was classified and evaluated by two classification methods. The first method was divided into chicken-wing, windsock, cactus, and cauliflower, and the second method was divided into one lobe, two lobes, and multiple lobes. The correlation between morphological feature of LAA and the recurrence rate of AF after RFA was analyzed.

**Results:** During follow-up, 12 patients (50%) and 10 patients (16.7%) had AF recurrence in persistent and paroxysmal AF, respectively. The LAA morphology was associated with the recurrence of AF after RFA with the chicken-wing highest recurrence risk (68.2%). The structure type of LAA was also related to the AF recurrence rate (*p* < 0.01). Compared with one lobe and multiple lobes, two lobes (recurrence, 47.6%) were more likely associated with the recurrence of AF (*p* < 0.02). Logistic regression analysis showed that the chicken-wing group had a higher risk of recurrence after RFA (OR = 8.13, *p* = 0.004), and the windsock group had a lower risk of recurrence (OR = 0.17, *p* = 0.002).

**Conclusion:** The morphological feature of LAA is related to the recurrence risk of AF after RFA. LAA morphology assessment can predict the risk of AF recurrence.

## Introduction

Atrial fibrillation (AF) is the most common cardiac arrhythmia. The incidence rate of AF is about 1–2% in the whole population (1) and increases with age; for those over 80 years of age, it is up to 9.1–11% (2). According to a retrospective study, the prevalence of AF in Asia was 0.1–4% and 2.8–14% in community and hospital surveys, respectively (3). When AF occurs, the left atrium autonomic pumping function is lost and the atrioventricular synergistic activity is impaired, which influences cardiac function and induced thrombosis. Until now, there have been many treatments for AF besides drug. Circumferential radiofrequency ablation (RFA) of the pulmonary vein is a safe and effective operation for AF (4). Usually, the success rate of RFA in treating paroxysmal AF is 80–90% after surgery, and persistent AF would be 60–80% (5–7). Indeed, the late recurrence rate of AF after a single RFA and repeated RFA are 11–29% and 7–24%, respectively (5–7). The leading cause of recurrence AF is incomplete ablation (8) and resumption of pulmonary vein-left atrial conduction (9).

Left atrial appendage (LAA) is a finger-like projection from the main body of the left atrium. The LAA protrudes from the primordial left atrium, which is formed mainly by the adsorption of the primordial pulmonary veins and their branches. The junction is well-defined by a narrowing at the orifice of the appendage. The orifice of LAA has few cardiomyocytes, while the body of LAA is rich (10). Therefore, the orifice becomes a potential conduction zone for reentry arrhythmia. There are considerable variations in its size, shape, and relationship with adjacent cardiac structure. Veinot et al. define lobes as protrusions from the main body with the tail portion representing a lobe, whereas bends in the tail do not constitute more lobes (10). Studies have shown that the LAA has one-lobe, two-lobe, and multiple-lobe structures Among Asians and Americans (11, 12). They found that two lobes were most common (54%), followed by three lobes (23%), one lobe (20%), and four lobes (3%) (13–16). Di Biase divides the LAA into four types: chicken-wing (48%), windsock (19%), cactus (30%), and cauliflower (3%) (17). The LAA also has a contractile function (18). The pathological state of AF causes the left atrial pressure to increase. The left atrium and the LAA can buffer the left atrial pressure by increasing the inner diameter and contraction force to ensure sufficient blood filling in the left ventricle. With AF progression, the LAA will be enlarged, which will reduce the blood flow and induce thrombosis in LAA.

Many studies have confirmed that the LAA has a stronger contraction and extension function than the left atrium (18, 19). It provides a theoretical basis for the LAA to be a source of AF and possibly affect prognosis. A limited study showed that the LAA volume was an independent factor for AF recurrence following RFA (20). This study focused on the relationship between LAA morphology and AF recurrence after RFA to predict the success rate of RFA.

## Methods

### Research Population

We retrospectively enrolled 84 patients (58 male/26 female) with AF in the Department of Cardiology, Shanghai East Hospital, from April 2014 to April 2018. Eighty-four patients were selected in this study, and 66 patients were lost to follow-up or could not tolerate medication.

Twenty-four patients with persistent AF and 60 patients with paroxysmal AF were performed by single RFA. Paroxysmal AF is defined as AF that terminates spontaneously or with intervention within 7 days of onset. Persistent AF is defined as AF that fails to self-terminate within 7 days. Moreover, all patients were examined by TEE before RFA to rule out LAA thrombus. Other exclusion criteria are age <20 years, pregnancy, history of cardiac surgery, valvular heart disease, congenital heart disease, thyroid dysfunction, severe liver and kidney dysfunction, electrolyte imbalance, and secondary AF. The study was approved by the hospital's institutional ethics committee, and written informed consent was provided by all patients.

### Transesophageal Ultrasonography

LAA morphology is defined by transesophageal ultrasonography based on the research (13, 17). The Philips IE33 color ultrasound diagnostic apparatus with multi-planar three-dimensional transesophageal probe X7-2t was used to detect the LAA morphology. The patients had fasted for 4–6 h before the examination, and then the oropharynx was anesthetized locally with 2% lidocaine. After that, the patient took a left lateral position. The probe was sent to the back of the left atrium, the cut surface angle was adjusted from 0 to 180°, and the probe was turned counterclockwise or clockwise to show the maximum LAA structure and thrombus (~135°). The pulse-wave sampling volume was then set in the LAA neck to obtain its flow, and the flow stream spectrum, and the image was stored.

### Definition of LAA Morphology

**Figure 1:**
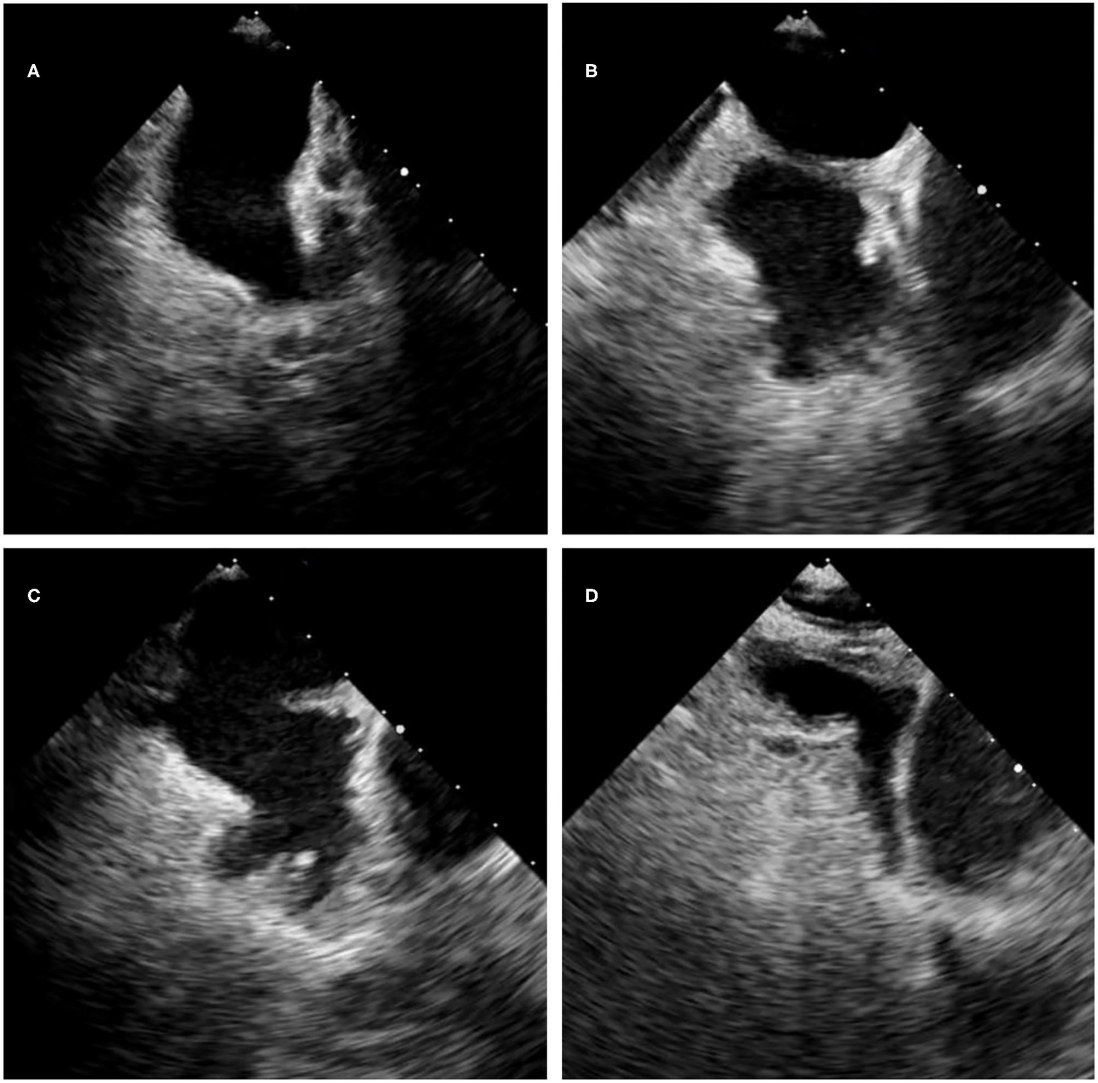
The LAA morphology by 2D transesophageal echocardiography. **(A)** The chicken-wing morphology. **(B)** The cauliflower morphology. **(C)** The cactus morphology. **(D)** The windsock morphology.

Here we displayed those LAA morphology by transesophageal echocardiography (Figure 1).The “chicken-wing” is described as having a dominant lobe that presents with an obvious bend in its proximal or middle part, folding back on itself at some distance from the orifice, and it may have secondary lobes. The “cactus” has a dominant central lobe and secondary lobes arise from it superiorly and inferiorly. The “cauliflower” has a short overall length, more complex internal characteristics, a variable number of lobes with lack of a dominant lobe, and a more irregular shape of the orifice. The “windsock” has a dominant lobe as the primary structure, and there are variations in the location and number of secondary or even tertiary lobes (21).

### Cardiac Catheter RFA

An endocardial map of the left atrium was created with an electroanatomic mapping system (CARTO, Biosense Webster, Irvine, CA, USA) and superimposed on the preexisting CMR image of the chamber. With routine hemodynamic and electrocardiographic monitoring, an ablation catheter (CARTO, Thermocool, Biosense Webster) was advanced to the left atrium. Circumferential lesions were applied surrounding the pulmonary veins. Additional ostial lesions were targeted to retain pulmonary vein potentials using a circular multipolar electrode-mapping catheter (Lasso, Biosense Webster). Entrance block into the pulmonary veins was confirmed in all patients as the primary procedural endpoint (22). In cases of persistent AF, the ablation procedure usually included additional ablation lesions, like CFAE ablation (23).

### Follow-Up and Assessment of AF Recurrence

All patients were followed up every 3 months at the outpatient clinic for 6–48 months after ablation. Twelve-lead ECGs or 24-h Holter monitoring was performed at 3, 6, and 12 months in clinic. In addition, 12-lead ECGs or 24-h Holter monitoring were also performed if patients reported palpitations. All patients were required to take oral anticoagulant medication (warfarin or new oral anticoagulants) for 2–3 months after RFA except contraindications. In cases of persistent AF, patients would receive oral amiodarone or propafenone for 3 months after surgery. Patients with paroxysmal AF did not need to use oral antiarrhythmic medications regularly. The blanking period is defined as a period post-ablation during which any recurrence of atrial arrhythmias is not considered a failure of the RFA. Here we set 3 months as blanking period according to the 2012 Consensus Statement on Catheter and Surgical Ablation of Atrial Fibrillation (24). According to the following criteria, recurrence of AF was defined: a documented AF episode of at least 30 s after the traditional blanking period.

### Statistical Analysis

Continuous variables were expressed as mean ± standard deviation, and categorical variables were expressed in frequency and percentage. The normal distributed continuous variable data were analyzed by *t*-test, and the skewness distributed data were compared by the *Mann–Whitney U*-test. All categorical variables were compared using the χ^2^-test or Fisher test (if the theoretical frequency is too small in the χ^2^-test, using the Fisher test). Multivariate logistic regression analysis was performed for variables with a *p*-value of < 0.05 in the univariate analysis. OR values and 95% confidence intervals (95% CI) were calculated to determine independent predictors associated with AF recurrence. Statistical analyses were performed using SPSS software, version 23 (SPSS Inc., Chicago, IL, USA). *p* < 0.05 indicated statistical significance.

## Results

### Subject Characteristics

A total of 84 patients were enrolled in the study. All patients received successful RFA. During the mean follow-up of 618.5 days [6 months−4 years], 22 (35%) patients experienced AF recurrence.

As Table 1 shows, there was a significant gender difference in postoperative AF recurrence. Males had a higher recurrence rate vs. females [19/58 (32.8%) vs. 3/26 (11.5%), *p* = 0.041]. Patients with persistent AF were more likely to relapse vs. paroxysmal AF patients [12/24 (50.0%) vs. 10/60 (16.7%), *p* = 0.002]. No significant difference was observed in age, BMI, preoperative blood pressure (systolic and diastolic blood pressure), and heart rate between the recurrent AF patients with the non-recurrent group. Although there were no differences between the two groups in the use of oral anticoagulants, ACEI or ARB, CCB, antiarrhythmics, and antiplatelet agents, the recurrent AF patients had a higher percentage using statin (recurrent AF vs. non-recurrent AF: 90.9 vs. 69.4%; *p* = 0.045). No differences were found between two groups in patients with hypertension, diabetes, stroke/TIA, coronary heart disease (postoperative PCI), myocardial bridge, and chronic obstructive pulmonary disease (COPD). We also did not observe any correlation of thyroid disease and obstructive sleep apnea syndrome with postoperative recurrence.

**Table 1:** Baseline characteristics.

	**AF recurrence**	**No AF recurrence**	***p*-value**
	**(*n* = 22)**	**(*n* = 62)**	
Sex (male) %	19 (86%)	39 (62.9%)	0.041
Age (years)	67.5 ± 7.5	66.1 ± 9.0	0.525
BMI	25.4 ± 9.0	26.2 ± 3.5	0.78
HBP %	14 (63.6%)	43 (69%)	0.622
DM %	5 (22.7%)	13 (21%)	0.54
**Other sign**			
Stroke/TIA	2 (9.1%)	2 (3.2%)	0.28
Atherosclerosis PCI	5 (22.7%)	10 (16.2%)	0.488
Myocardial bridge	0	1 (0.02%)	0.738
COPD	1 (0.05%)	1 (0.02%)	0.458
GU	0	1 (0.02%)	0.738
Thyroid disease	2 (9.1%)	1 (0.02%)	0.166
OSAS	1 (0.05%)	0	0.262
Time (months)	26.8 ± 34.1	32.6 ± 45.1	0.534
**Type**			
Persistent AF	12 (54.5%)	12 (19.4%)	0.002
Paroxysmal AF	10 (45.5%)	50 (80.6%)	
With AFL	2	11	0.335
**BP (mmHg)**			
SBP	130.6 ± 23.3	134.5 ± 18.9	0.438
DBP	80.0 ± 11.8	85.1 ± 22.2	0.313
HR (cpm)	77.4 ± 20.2	86.3 ± 23.2	0.113
**Devices/medication**			
NOACs	8 (36.4%)	32 (51.6%)	0.219
VKAs	14 (63.6%)	30 (48.4%)	
Anti-platelet	2 (9.1%)	4 (6.5%)	0.65
I anti-arrhythmia	7 (31.8%)	12 (15.4%)	0.336
II anti-arrhythmia	5 (22.7%)	15 19.2%)	0.712
III anti-arrhythmia	14 (63.6%)	39 (50%)	0.609
ACEI or ARB	13 (59.1%)	36 (58.1%)	0.933
CCB	4 (18.2%)	19 (30.6%)	0.26
Statins	20 (90.9%)	43 (69.4%)	0.045

*AF, atrial brillation; TIA, transient ischemic attack; BMI, body mass index; HBP, high blood pressure; DM, diabetes mellitus; COPD, chronic obstructive pulmonary disease; GU, gastric ulcer; OSAS, obstructive sleep apnea-hypopnea syndrome; AFL, atrial flutter; SBP, systolic blood pressure; DBP, diastolic blood pressure; HR, heart rate; NOACs, novel oral anticoagulants; VKAs, vitamin K antagonists; ACEI, angiotensin-converting enzyme inhibitor; ARB, angiotensin-II receptor blocker; CCB, calcium channel blocker*.

Routine preoperative lab tests for patients were ordered, and the results are shown in Table 2. There was no difference between the recurrence and no recurrence groups.

**Table 2:** Laboratory measurements.

	**AF recurrence**	**No AF recurrence**	***p*-value**
	**(*n* = 22)**	**(*n* = 62)**	
PRO-BNP	745.1 ± 1,000.7	793.9 ± 878.3	0.447
**Myocardial enzyme**			
Troponin (ng/ml)	0.015 ± 0.013	0.015 ± 0.016	0.47
Myoglobin (ng/ml)	45.5 ± 27.0	40.0 ± 23.2	0.328
Creatine kinase isoenzyme (ng/ml)	5.9 ± 12.1	2.2 ± 1.7	0.5
**Renal function**			
Creatinine (μmol/l)	89.0 ± 24.3	80.6 ± 19.5	0.111
Carbamide (mmol/l)	9.7 ± 10.9	6.0 ± 1.8	0.212
Purine trione (μmol/l)	380.7 ± 143.5	331.3 ± 113.9	0.109
**Blood lipid**			
TC (mmol/l)	3.4 ± 0.8	4.2 ± 1.2	0.09
TG (mmol/l)	1.4 ± 0.9	1.5 ± 0.9	0.35
LDL (mmol/l)	1.98 ± 0.66	2.59 ± 0.91	0.375
HDL (mmol/l)	1.2 ± 0.4	1.3 ± 0.6	0.651
HCY	10.40 ± 1.96	12.12 ± 5.06	0.385
**Liver function**			
AAT (μ/l)	127.4 ± 7.5	113.8 ± 45.5	0.529
ALT (μ/l)	23.96 ± 20.09	32.44 ± 22.22	0.245
AST (μ/l)	23.5 ± 17.1	30.7 ± 28.2	0.635
γ-GT (μ/l)	33.96 ± 31.27	36.93 ± 30.08	0.802
TBIL (μmol/l)	19.0 ± 27.4	13.9 ± 6.4	0.36
DBIL (μmol/l)	5.9 ± 2.7	6.3 ± 3.0	0.721
IBIL (μmol/l)	7.2 ± 3.9	7.5 ± 4.2	0.386
FBG	5.89 ± 1.14	6.28 ± 1.81	0.559
**Thyroid function**			
T4 (μg/dl)	7.64 ± 1.37	7.43 ± 1.25	0.536
FT4 (ng/dl)	1.37 ± 0.17	1.34 ± 0.21	0.502
TSH (μIU/ml)	2.1 ± 1.6	3.1 ± 4.7	0.374
T3 (ng/ml)	1.08 ± 0.32	1.06 ± 0.24	0.568
FT3 (pg/ml)	3.13 ± 0.95	2.92 ± 0.37	0.346
**Inflammation**			
CRP (mg/l)	5.27 ± 6.97	6.99 ± 11.93	0.406
WBC (*109/l)	5.9 ± 1.3	6.7 ± 1.9	0.054
N% (%)	64.6 ± 8.5	64.5 ± 13.0	0.498

*PRO-BNP, proB-type natriuretic peptide; TC, total cholesterol; TG, triglycerides; LDL, low-density lipoprotein; HDL, high-density lipoprotein; HCY, homocysteine; AAT, alpha antitrypsin; ALT, alanine aminotransferase; AST, aspartate transaminase; TBIL, total bilirubin; DBIL, direct bilirubin; IBIL, indirect bilirubin; T4, tetraiodothyronine; FT4, free thyroxine; TSH, thyroid-stimulating hormone; T3, triiodothyronine; FT3, free triiodothyronine; CRP, C-reactive protein; WBC, white blood cell; N%, neutrophil%*.

### The Association of LAA Morphology With AF Recurrence

**Figure 2:**
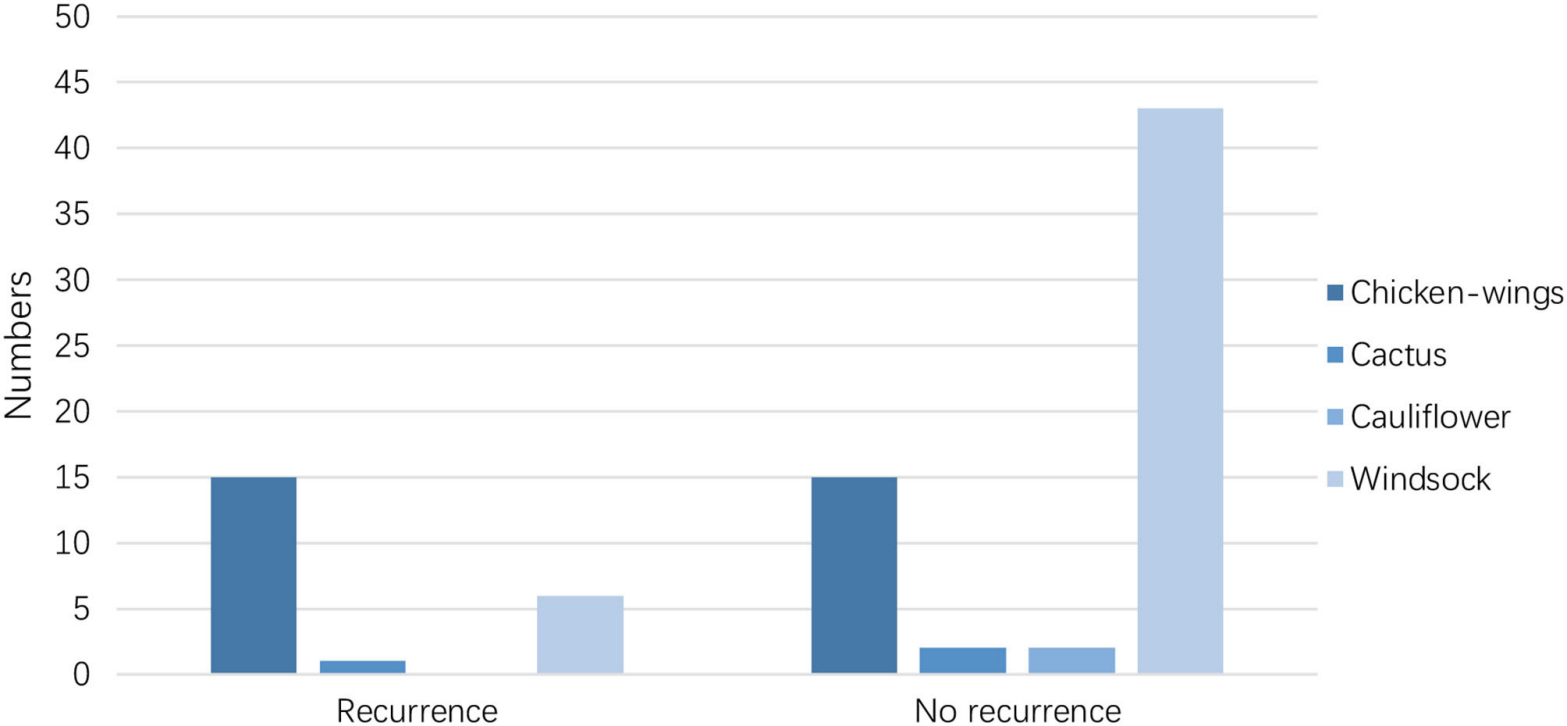
LAA morphology with AF recurrence.

**Figure 3:**
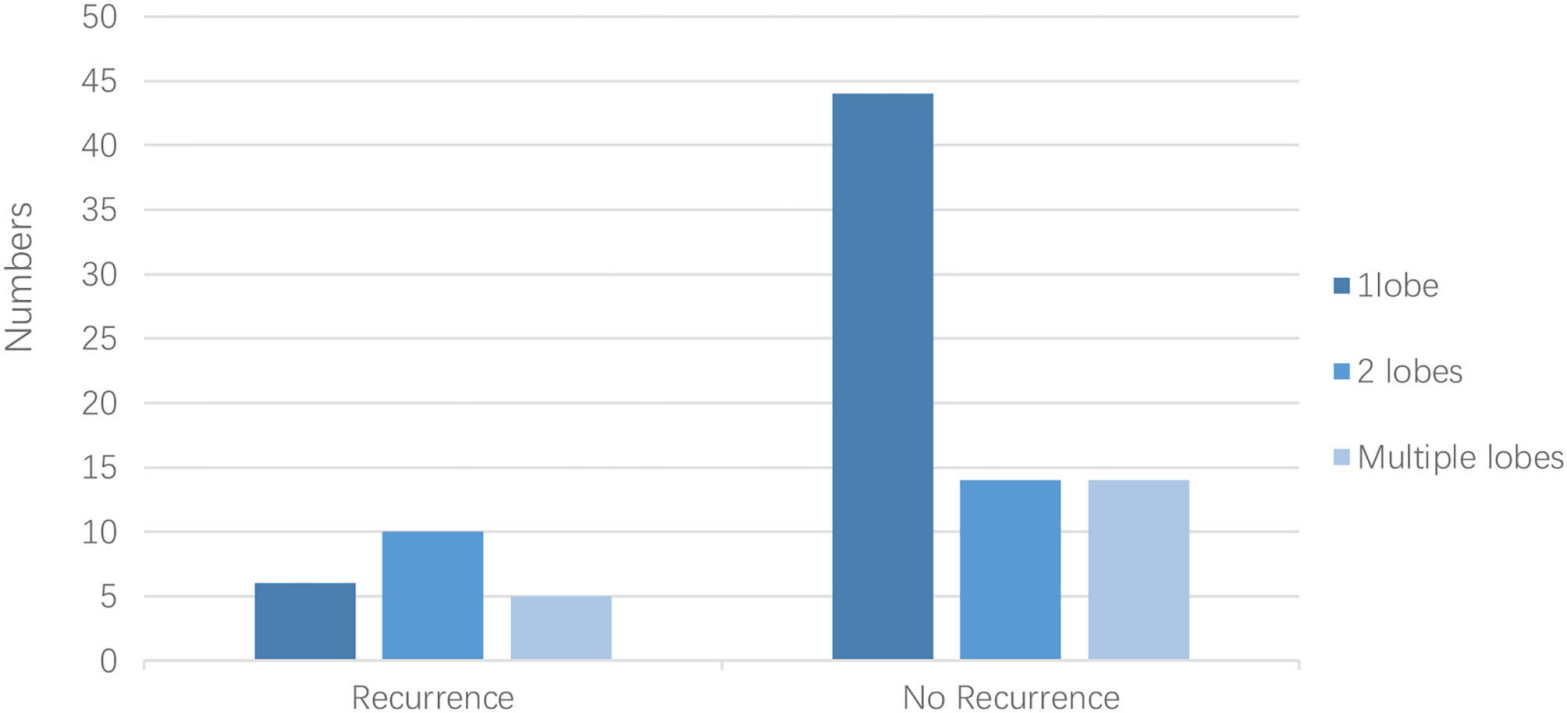
LAA structure (lobes) with AF recurrence.

The LAA morphology is shown in Table 3 and Figure 2. The Fisher test results showed a significant difference in AF recurrence among four types of LAA (*p* = 0.001). Among the four types, the risk of AF recurrent in chicken-wing LAA was higher than in the windsock (*p* < 0.013) after the adjusted Bonferroni *p*-value. There was an insignificant difference among the others (*p* > 0.05). The LAA structure (lobes) was associated with recurrence (*p* = 0.008) (Table 4 and Figure 3), and the multiple lobes had more risk of recurrence of AF than the one lobe (*p* < 0.016).

**Table 3:** LAA morphology with AF recurrence.

	**LAA morphology**					***p*-value**
	**Chicken-wings**	**Cactus**	**Cauliflower**	**Windsock**	**Total**	
Recurrence	15	1	0	6	22	0.001
No recurrence	15	2	2	43	62	
Total	30	3	2	49	84	

*p-value for the differences between the 4 LAA morphology groups*.

**Table 4:** LAA structure (lobes) with AF recurrence.

	**LAA structure (lobes)**				***p*-value**
	**1 lobe**	**2 lobes**	**Multiple lobes**	**Total**	
Recurrence	7	5	10	22	0.008
No recurrence	44	14	14	62	
Total	51	24	19	84	

*p-value for the differences between the three LAA structure groups. The group of recurrence has one data missed*.

### The Association of Ablation Strategy With AF Recurrence

All the patients experienced those ablation strategies: PVAI, PVAI+1Lines, PVAI+CFAE, or PVAI+3 Lines+CFAE; there was no significant difference among the four types of surgery by the χ^2^-test (*p* = 0.07, Table 5).

**Table 5:** The relation between ablation strategy and AF recurrence.

	**Ablation strategy**					***p*-value**
	**PVAI**	**PVAI+1 lines^a^**	**PVAI+CFAE^b^**	**PVAI+3 lines+CFAE**	**Total**	
Recurrence	9	2	5	6	22	0.07
No recurrence	40	8	9	5	62	
Total	48	11	14	12	84	

a *One line (cavo-tricuspid isthmus line); three lines (mitral isthmus line, LA roof line, cavo-tricuspid isthmus line)*.

b *CFAE, complex fractionated atrial electrograms*.

### The Association of LAA Function With AF Recurrence

TEE showed an insignificant correlation between the AF recurrent group and non-AF recurrent group in LAA max/min area, max/min orifice diameter, flow emptying velocity, and flow filling velocity (Table 6).

**Table 6:** Parameters of LAA from TEE in patients with and without AF recurrence.

**Rat echocardiography**	**AF recurrence (*n* = 22)**	**No AF recurrence (*n* = 62)**	***p*-value**
Maximum area of LAA (cm^2^)	4.13 ± 1.89	4.12 ± 1.50	0.218
Minimum area of LAA (cm^2^)	1.53 ± 0.98	1.36 ± 0.80	0.309
LAA-FEV (cm/s)	50.52 ± 23.86	52.65 ± 22.07	0.695
LAA-FFV (cm/s)	50.96 ± 21.09	56.03 ± 23.32	0.515
The max orifice diameter of LAA (mm)	2.75 ± 0.61	2.79 ± 0.56	0.821
The min orifice diameter of LAA (mm)	2.00 ± 0.54	1.80 ± 0.39	0.066

### Univariate and Multivariate Logistic Regression of the AF Recurrence After RFA

The type of AF (*p* = 0.003), LAA morphology (*p* = 0.0005), and LAA structure (*p* = 0.007) had statistical significance in univariate logistic regression (Table 7). Multivariate logistic regression is shown in Table 8. There was no significant correlation between AF recurrence and sex, type of AF, statin use, ablation strategy, and LAA function. In the classification of LAA, chicken-wing and windsock LAA were significantly associated with postoperative recurrence. The risk of AF recurrence after RFA in the chicken-wing group was higher than in other groups. The chicken-wing LAA was a risk factor for AF recurrence after RFA [OR = 8.13 (1.94–34.02)]. The AF recurrence of the windsock LAA group was lower than that of the other group [OR = 0.17 (0.06–0.51)]. There was an insignificant difference between cactus and cauliflower LAA [OR = 0.34 (0.01–14.38), and OR = 0.53 (0.04–6.51)]. In the LAA structure (lobe) classification, the risk of recurrence in one lobe was lower than in other structures [OR = 0.12 (0.02–0.56)], and there was no statistically significant difference in the recurrence of AF between the multiple lobes and the two lobes.

**Table 7:** Univariate logistic regression analysis for variables with AF recurrence.

	**Odds ratio (95% confidence interval)**	***p*-value**
Male sex	0.268 (0.071–1.004)	0.051
Type of AF (persistent)	5 (1.751–14.280)	0.003
Use of statins	0.226 (0.048–1.067)	0.06
Age	1.006 (0.950–1.065)	0.843
Maximum area of LAA	0.986 (0.876–1.110)	0.817
Minimum area of LAA	1.206 (0.817–1.779)	0.346
LAA-FEV	0.994 (0.972–1.017)	0.618
LAA-FFV	0.991 (0.968–1.014)	0.442
The max orifice diameter of LAA	0.867 (0.362–2.073)	0.748
The min orifice diameter of LAA	2.725 (0.881–8.426)	0.082
LAA morphology	0.513 (0.353–0.746)	0.0005
LAA structure	2.137 (1.235–3.696)	0.007
Ablation strategy	1.704 (1.112–2.611)	0.095

**Table 8:** Logistic regression analysis for variables with AF recurrence.

	**Odds ratio (95% confidence interval)**	***p*-value**
Male sex	4.49 (0.73–27.85)	0.106
Type of AF (persistent)	0.32 (0.07–1.39)	0.128
Use of statins	3.76 (0.59–24.16)	0.163
Age	0.916 (0.810–1.037)	0.165
Ablation strategy	0.328 (0.142–0.755)	0.090
Maximum area of LAA	2.082 (0.861–5.032)	0.104
Minimum area of LAA	0.310 (0.059–1.623)	0.166
LAA-FEV	0.980 (0.907–1.059)	0.610
LAA-FFV	1.015 (0.940–1.097)	0.702
The max orifice diameter of LAA	3.553 (0.408–30.917)	0.251
The min orifice diameter of LAA	0.187 (0.022–1.612)	0.127
**LAA morphology**		
Chicken-wing	8.13 (1.94–34.02)	0.004
Cactus	0.34 (0.01–14.38)	0.574
Cauliflower	0.53 (0.04–6.51)	0.622
Windsock	0.17 (0.06–0.51)	0.002
**LAA structure**		
1 lobe	0.12 (0.02–0.56)	0.007
2 lobes	0.80 (0.17–3.80)	0.779
Multiple lobes	3.45 (0.18–66.44)	0.411

### Kaplan–Meier Survival Curves of AF Recurrence After RFA

**Figure 4:**
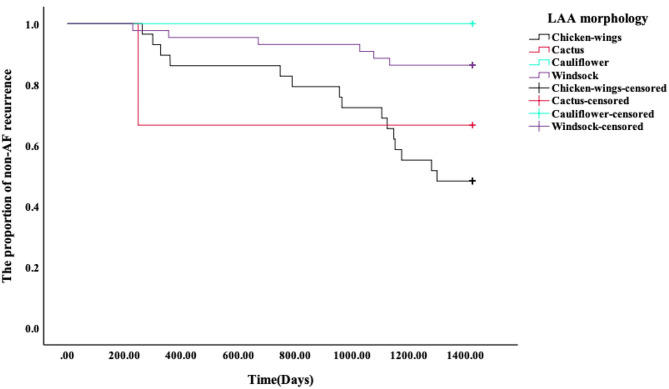
Kaplan–Meier analysis for recurrence after RFA. The Kaplan–Meier survival curves of AF recurrence after ablation and the log-rank test between the four types' LAA morphology during 1,460 days of follow-up.

**Figure 5:**
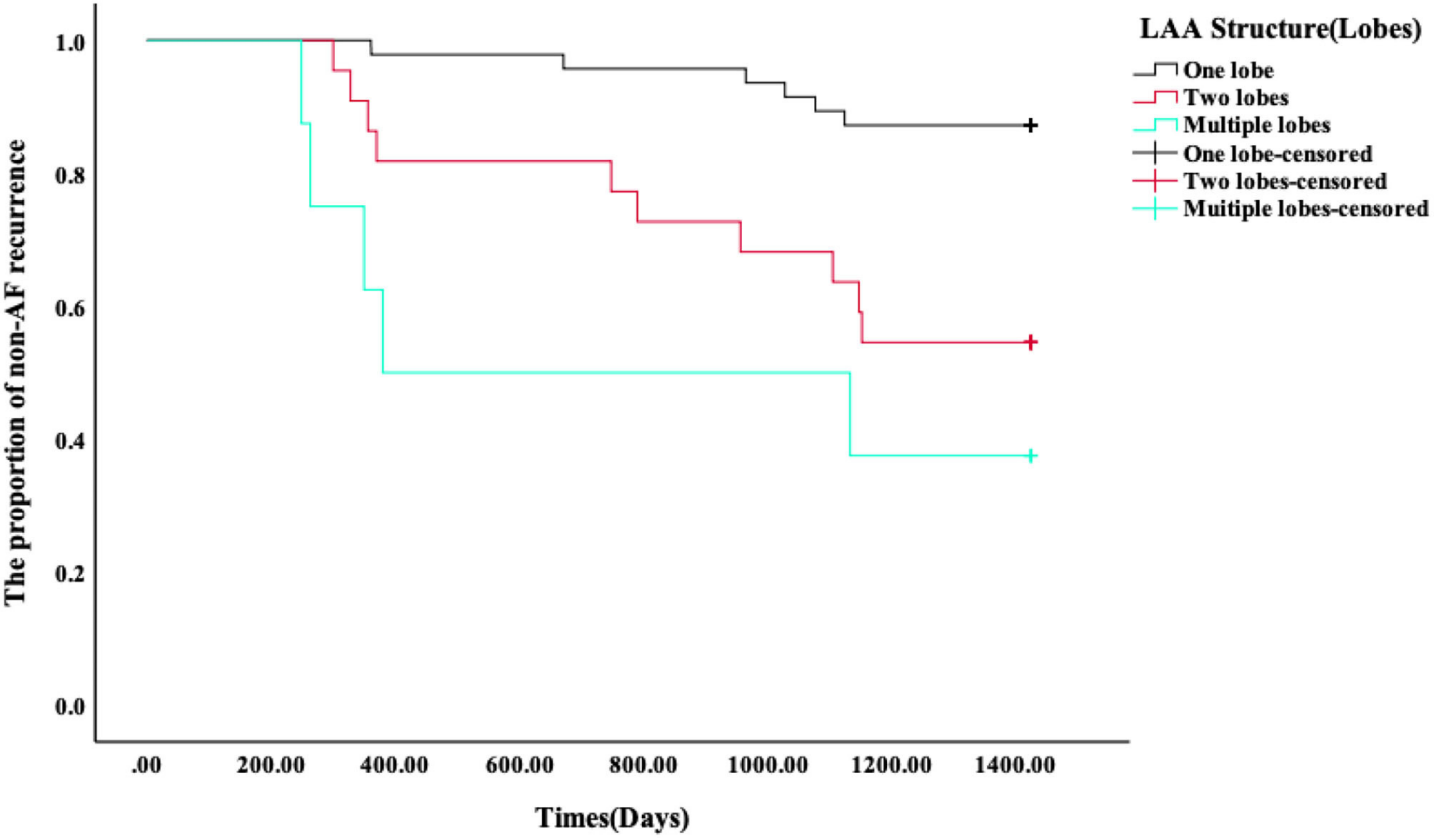
Kaplan–Meier analysis for recurrence after RFA. The Kaplan–Meier survival curves of AF recurrence after ablation and the log-rank test between the three different lobes during 1,460 days of follow-up.

Kaplan–Meier survival curves showed that the AF recurrence rate after ablation was significantly different between the four different LAA morphology (Figure 4, *p* < 0.01) during the 1,460 days follow-up, the LAA morphology of chicken wings were more likely to have a high proportion of AF recurrence. Kaplan–Meier survival curves also showed that the proportion of AF recurrence was significantly different between the three different lobes (Figure 5, *p* = 0.01); the AF recurrence rate was high in the multiple-lobe group.

## Discussion

Our research found that the morphological structure of LAA is associated with AF recurrence after RFA. The chicken-wing shape is a risk factor for postoperative AF recurrence. The one-lobe type is less likely to cause AF recurrence compared with other types. We speculate that the main reason may be related to anatomy and electrophysiology. The bend angle of chicken-wing LAA is larger and longer, and some of them have multiple distal lobes, making deeper and more complex electrophysiological activities, leading to easy recurrence after ablation. It is also explained that one-lobe LAA have a low recurrence rate. During the development of the left atrium, the LAA is considered to be an unimportant accessory structure. However, as the research progresses, the understanding of the LAA becomes more and more profound. Di Biase et al. report that LAA isolation could prevent secondary RFA caused by AF recurrence (25). Studies have shown that 27% of patients have an LAA local ectopic pacemaker after pulmonary vein isolation, and in 8.7% of patients, LAA is the only source of recurrent arrhythmias (26). Ablation of the LAA's anterior wall near the LAA's neck and prolonging or electrically isolating the LAA can effectively prevent AF recurrence (18). These studies suggested that LAA is an essential factor for the recurrence of AF and the structure or the morphology of LAA may influence the recurrence of AF. Our study confirmed that LAA morphology can influence AF recurrence. The first reason is that the contraction and extension of LAA are stronger than those of the left atrium, and it plays a buffering role in decreasing left atrial pressure (27, 28). Secondly, the Bachmann beam and Marshall ligament near the LAA are the main conduction pathways of atrial electrical activity. The efferent fibers of the LAA sympathetic and vagus nerves are essential for maintaining the regular electrophysiological activity of the LAA. The left atrium with different structures of LAA may have different electrophysiological activities. Lastly, the LAA is a well-known source of atrial natriuretic peptide (ANP). It is associated with B-type natriuretic peptide levels (BNP), adiponectin, insulin, and free fatty acids. Studies have shown that surgical epicardial LAA closure can lead to downregulation of the adrenergic system and RAAS (29, 30).

Balk et al. (31) confirmed that patients with non-paroxysmal AF had an average of 60% higher probability of AF recurrence after RFA than patients with paroxysmal AF. This finding is consistent with our research. However, any individual factor or a set of patient's underlying eigenvalues do not consistently and independently predict the recurrence of AF, possibly due to a particular relationship and interaction between the patient's disease characteristics and cardiac measurements.

In this study, statins were associated with AF recurrence after RFA in univariate analysis. However, multivariate regression analysis did not yield any positive association positive results. Peng et al. (32) studied the relationship between statins and the recurrence rate of AF after RFA, indicating that statins have no advantage in reducing AF recurrence after AF ablation. However, in a subgroup analysis, statins were associated with a lower AF recurrence rate after RFA. More randomized controlled trials are needed to confirm it. Statins have anti-inflammatory and antioxidant actions that may act on atrial structural and electrical remodeling (31). Atorvastatin can inhibit ROS production by downregulating NOX2 and alleviating atrial fibrosis, thereby preventing AF (32). For these reasons, we infer that statins may have an effect of preventing AF recurrence after catheter ablation.

In the current study, we explored some risk factors of AF recurrence after RFA. We found that the morphology of the LAA, including morphology (shape) and structure (lobes), is associated with postoperative recurrence, which may be associated with different electrical conductivities and structural specificities of LAA. The TEE can measure the shape and structure of LAA. It will enable us to understand better LAA morphology and the relationship between the LAA morphology and AF recurrence.

## Limitations

First, this is a small-sample, retrospective study, because there is a limited number of patients who had TEE. Prospective research with more samples is needed to confirm the results. Secondly, the AF recurrence after RFA is a combination of the related factors, including genetic polymorphism, inflammatory factors, and left atrial fibrosis. The associated basic research investigation will clarify the mechanism, and the basic research and clinical combination need to be further studied.

## Conclusions

The morphological structure of LAA is one of the causes of AF recurrence after RFA. The chicken-wing LAA is a risk factor for postoperative AF recurrence. The risk of AF recurrence in windsock LAA is lower than that of others. Compared with the multiple-lobe type, the one-lobe type is less likely to cause AF recurrence. A better understanding of the LAA morphology before RFA of AF contributes to predicting postoperative AF recurrence risk.

## Data Availability Statement

The original contributions presented in the study are included in the article/supplementary material, further inquiries can be directed to the corresponding author/s.

## Ethics Statement

Written informed consent was obtained from the individual(s) for the publication of any potentially identifiable images or data included in this article.

## Author Contributions

XZ and SG contributed to conception and design of the study. XM and YC organized the database. XZ and JZ performed the RFA of AF. YG performed the transesophageal echocardiography. SG and BL wrote the manuscript. All authors contributed to manuscript revision, read, and approved the submitted version.

## Conflict of Interest

The authors declare that the research was conducted in the absence of any commercial or financial relationships that could be construed as a potential conflict of interest.

## Publisher's Note

All claims expressed in this article are solely those of the authors and do not necessarily represent those of their affiliated organizations, or those of the publisher, the editors and the reviewers. Any product that may be evaluated in this article, or claim that may be made by its manufacturer, is not guaranteed or endorsed by the publisher.
